# Supplementary material for: Mycobacterium tuberculosis exploits host ATM kinase for survival advantage through SecA2 secretome
Source: eLife. 2020 Mar 30;9:e51466. doi: 10.7554/eLife.51466 (PMC7162654; doi:10.7554/eLife.51466)
Supplement: Transparent reporting form [file elife-51466-transrepform.doc]

***eLife’s* transparent reporting form**

We encourage authors to provide detailed information *within their submission* to facilitate the interpretation and replication of experiments. Authors can upload supporting documentation to indicate the use of appropriate reporting guidelines for health-related research (see [EQUATOR Network](http://www.equator-network.org/ )), life science research (see the [BioSharing Information Resource](https://biosharing.org/)), or the [ARRIVE guidelines](http://www.plosbiology.org/article/info:doi/10.1371/journal.pbio.1000412) for reporting work involving animal research. Where applicable, authors should refer to any relevant reporting standards documents in this form.

If you have any questions, please consult our Journal Policies and/or contact us: [editorial@elifesciences.org](mailto:editorial@elifesciences.org).

**Sample-size estimation**

- You should state whether an appropriate sample size was computed when the study was being designed
- You should state the statistical method of sample size computation and any required assumptions
- If no explicit power analysis was used, you should describe how you decided what sample (replicate) size (number) to use

Please outline where this information can be found within the submission (e.g., sections or figure legends), or explain why this information doesn’t apply to your submission:

Sample size in Fig 7a uninfected (n=4) and *Rv (n=4 at day1; n=5 at day 56)*.

Sample size in Fig 7e & 7f *Rv (n=5 at day1; n=6 and n=6 at day 15 and 30)*.

**Replicates**

- You should report how often each experiment was performed
- You should include a definition of biological versus technical replication
- The data obtained should be provided and sufficient information should be provided to indicate the number of independent biological and/or technical replicates
- If you encountered any outliers, you should describe how these were handled
- Criteria for exclusion/inclusion of data should be clearly stated
- High-throughput sequence data should be uploaded before submission, with a private link for reviewers provided (these are available from both GEO and ArrayExpress)

Please outline where this information can be found within the submission (e.g., sections or figure legends), or explain why this information doesn’t apply to your submission:

When we state biological replicate, we mean experiments performed at different times. When we state technical replicates, we mean experiments performed at same time but processed separately.

Experiments to determine CFUs in Fig 3b, 5c, 5e, 6c, 6f, 7c were performed in three biological replicates with three technical replicates each time.

CFU graphs shown are representative of one biological experiment performed in technical triplicates.

Error bars represent standard deviation (SD). Significance was calculated with students t test, 2 tailed, homoscedastic in Microsoft Excel.

Fig 6g (apoptosis-Annexin V staining) experiment has been analyzed through three biological replicates. Error bars represent standard deviation (SD). Significance was calculated with students t test, 2 tailed, homoscedastic in Microsoft Excel.

Densitometric analysis of important western blots (Figure Supplement 7) was performed using Image J software. Significance was calculated from three biological replicates of blots.

**Statistical reporting**

- Statistical analysis methods should be described and justified
- Raw data should be presented in figures whenever informative to do so (typically when N per group is less than 10)
- For each experiment, you should identify the statistical tests used, exact values of N, definitions of center, methods of multiple test correction, and dispersion and precision measures (e.g., mean, median, SD, SEM, confidence intervals; and, for the major substantive results, a measure of effect size (e.g., Pearson's r, Cohen's d)
- Report exact p-values wherever possible alongside the summary statistics and 95% confidence intervals. These should be reported for all key questions and not only when the p-value is less than 0.05.

Please outline where this information can be found within the submission (e.g., sections or figure legends), or explain why this information doesn’t apply to your submission:

Error bars represent standard deviation (SD). Significance was calculated with students t test, 2 tailed, homoscedastic in Microsoft Excel. 95% confidence interval. *, p≤0.05; **, p≤0.005; ***, p≤0.0005. p value where needed have not been indicated if fall below 0.05.

(For large datasets, or papers with a very large number of statistical tests, you may upload a single table file with tests, Ns, etc., with reference to sections in the manuscript.)

**Group allocation**

- Indicate how samples were allocated into experimental groups (in the case of clinical studies, please specify allocation to treatment method); if randomization was used, please also state if restricted randomization was applied
- Indicate if masking was used during group allocation, data collection and/or data analysis

Please outline where this information can be found within the submission (e.g., sections or figure legends), or explain why this information doesn’t apply to your submission:

No experiment which need group allocation has been shown.

**Additional data files (“source data”)**

- We encourage you to upload relevant additional data files, such as numerical data that are represented as a graph in a figure, or as a summary table
- Where provided, these should be in the most useful format, and they can be uploaded as “Source data” files linked to a main figure or table
- Include model definition files including the full list of parameters used
- Include code used for data analysis (e.g., R, MatLab)
- Avoid stating that data files are “available upon request”

Please indicate the figures or tables for which source data files have been provided:

***Figure 3: Source Data 1: . Intracellular Mtb causes continuous DSBs.*** Source data for Figure 3b.

***Figure 5. Source Data 2: ATM activation provides survival advantages to Mtb.***  Source Data for Figure 5c and 5e.

***Figure 6. Source Data 3: ATM inhibition hypersensitizes host cells to apoptosis.*** Source Data for Figures 6c, 6f and 6g.

***Figure 7: Source Data 4. ATM kinase is a potential therapeutic target for host directed TB therapy.***  Source Data for Figure 7a, 7c, 7e and 7f.
